# Genes *versus* Environment: cytoplasmic BAP1 determines the toxic response to environmental stressors in mesothelioma

**DOI:** 10.1038/cddis.2017.293

**Published:** 2017-06-29

**Authors:** Ivano Amelio

**Affiliations:** 1 Medical Research Council, Toxicology Unit, Leicester, UK

The dissection of the interaction nodes between genome and environment is a long-standing challenge in biomedical research, and how much these two components individually contribute to human health remains currently elusive. The BRCA1-associated protein 1 (BAP1) is an example of a critical player in determining the outcome of the interaction of Genes *versus* Environment and two recent studies coordinated by a collaboration of the Carbone, Pinton, Yang and Jia groups provide a mechanistic rationale for the contribution of BAP1 to carcinogen sensitivity.^1, 2^

BAP1 is an ubiquitin C-terminal hydrolase belonging to a larger group of deubiquitylating enzymes.^3^ Original studies by Carbone’s group identified the association of germline mutations in *BAP1* with early onset of benign melanocytic skin tumours and a later high incidence of malignant mesothelioma together with uveal and cutaneous melanoma.^4, 5, 6^ The study conducted on two unrelated US families, referred to as ‘L’ and ‘W’ for their origins from Louisiana and Wisconsin respectively, implicated the 3p21 chromosomal region with an unexpectedly high incidence of malignant mesothelioma following minimal exposure to asbestos.^2^ Sequencing of this locus led to the identification of heterozygous mutations in BAP1, uncovering a novel familial autosomal dominant cancer syndrome. Indeed, biallelic somatic mutations in BAP1 are also frequently observed in human cancers.^7, 8^ Nearly all cancer types have been reported in carriers of BAP1 germline mutations, but the high incidence of tumours associated with environmental stressors, such as mesothelioma (with asbestos), melanoma and skin tumours (with UV), highlights BAP1 as a critical player in the interaction of genes with environment.

Despite the critical relevance of BAP1 for cancer predisposition, the mechanistic insights underlining its function have so far been elusive. Originally identified in a yeast two‐hybrid screen as an interactor of the RING finger domain of the tumour suppressor BRCA1, BAP1 was eventually disproved as a BRCA1 deubiquitylating enzyme.^3^ Nuclear BAP1 indeed deubiquitylates BRCA1‐associated RING domain 1 (BARD1), which in a heterodimer with BRCA1, forms the E3 ubiquitin ligase that regulates the DNA damage response (DDR).^9, 10^ Loss of BAP1 resembles BRCA1 deficiency, resulting in hypersensitivity to ionising radiation.^3^ However, an important unexplored aspect of BAP1 is the significant proportion of the protein that can be sequestered in the cytoplasm by multi-monoubiquitylation of its nuclear localisation signal. In this study Bononi *et al.* uncovered a cytoplasmic function of BAP1, implicating a novel mechanism for the contribution of BAP1 loss to environmental carcinogenesis.

The authors employed an elegant experimental approach, using BAP1^+/^^−^ and BAP1^+/+^ fibroblasts derived from carriers and control members of families ‘W’ and ‘L’. BAP1^+/^^−^ fibroblasts express 50% BAP1 protein levels as compared to control fibroblasts, and represent an excellent cellular model to experimentally recapitulate BAP1 deficiency in pre-carcinogenic conditions. In the work published in *Nature*^1^ the authors showed in subcellular fractionation experiments a significant accumulation of BAP1 in the cytoplasm and more specifically in the endoplasmic reticulum (ER). A reduced level of BAP1 in BAP1^+/−^ fibroblasts mainly affected the ER fraction of the protein, suggesting a potential dysfunction of the ER in the mutant cells.^1^ As BAP1^+/−^ cells appeared hyposensitive to a wide range of pro-apoptotic drugs and inducers, the authors speculated that BAP1 may contribute to control of the pro-apoptotic Ca^2+^ release from the ER. BAP1^+/−^ fibroblasts indeed displayed a reduced level of Ca^2+^ release from the ER, which was restored by re-expression of chimeric BAP1 mutants selectively targeting ER and cytoplasm, but not the nucleus. Thus, Carbone *et al.* reported an unanticipated cytoplasmic function of BAP1 that could explain its critical role in determining the biological outcome of its interaction with the environmental stressors.^1^

As a ubiquitin hydrolase, BAP1 exerts an enzymatic activity which directly influences stability and activation of its substrates. Investigating the cytoplasmic function of BAP1, the authors focused on the possible interaction between BAP1 and ER channels that control Ca^2+^ flux to the cytoplasm. The data clearly indicated that BAP1 physically interacts with the type-3 inositol-1,4,5-trisphosphate-receptor (IP3R3), and BAP1 expression level is directly correlated with IP3R3 in wild-type and mutant fibroblasts. Deeper biochemical analysis indeed demonstrated that BAP1 enzymatically controls IP3R3 ubiquitination and thus positively influences its stability. Loss of BAP1 therefore results in reduced expression of IP3R3 with correspondingly reduced cytoplasmic Ca^2+^ release, which in turn determines resistance to apoptotic inducers. The authors also observed an increase of cytoplasmic Ca^2+^ release and apoptosis in crocidolite asbestos-treated primary human mesothelial cells. Remarkably, depletion of BAP1 or IP3R3 promoted crocidolite asbestos/TNF-α-induced transformation of primary mesothelial cells, and re-expression of IP3R3 in BAP1-depleted cells partially reverted the phenotype.^1^ However, experiments with subcellular specific chimeric mutants of BAP1 indicated that both nuclear and cytoplasmic BAP1 contributed to the phenotype. This latter data opens the question regarding the specific contribution of the balance between cytoplasmic and nuclear BAP1 to cell death and genomic stability upon interaction with environmental stressors.

A second study published in *Cell Death & Differentiation*^2^ by the same groups reports an intriguing observation in BAP1^+/−^ individuals, which might shed light on the molecular basis of the transformation potential of these cells.^2^ By metabolic profiling of fibroblasts and plasma of carriers and control members from ‘W’ and ‘L’ families, the authors identified a clear alteration in cellular metabolism adopted by the mutant cells. BAP1^+/−^ fibroblasts displayed a typical ‘Warburg effect’, as their cellular metabolism largely relied on aerobic glycolysis, increased lactate production and reduced mitochondrial respiration.^2^ Metabolic profiling of plasma from carrier individuals also displayed a similar distribution of the metabolites, and their profile was predictive of the genotype with 100% accuracy. The originality of these data relies on the observation of metabolic rewiring typical of transformed cells in a premalignant cellular setting, thus suggesting that that ‘Warburg effect’ might facilitate cancer transformation rather than be an adaptive process occurring in malignancy. Whether the observed metabolic change is associated with alteration of the Ca^2+^ flux and ER/mitochondria function of BAP1^+/−^ cells will require further investigation and will probably highlight additional layers of complexity on the contribution of BAP1 deficiency to carcinogen hypersensitivity.

In conclusion, these studies have clarified novel important molecular aspects associated with the cancer predisposition of BAP1^+/−^ carrying individuals and suggest new relevant questions, related to BAP1-dependent cancer susceptibility, and to more general gene/environment interactions (Figure 1). It is now important to fully dissect the relative contribution of cytoplasmic *versus* nuclear BAP1 to the different aspects of carcinogenesis. In line with this, additional molecular details are required in relation to the control of BAP1 function, as, for example, whether and which (micro)-environmental signals can trigger BAP1 subcellular localisation and consequentially balance its contribution to genomic stability and cell death. Also, it will be important to assess the contribution of BAP1 somatic mutations to the tumorigenic process, and whether their incidence correlates with a selective mutational landscape. In addition, the contribution of premalignant metabolic rewiring to tumour transformation will stimulate a broad interest in the field. This aspect might also have wider implications for the genes−environment interaction, as it might imply that dietary regimes could influence metabolism and cellular transformation in specific predisposed conditions such as BAP1 mutations. Finally, these studies further highlight the importance of the integration of multidisciplinary approaches to dissect the interactions between genetic and environmental factors, to translate this information and stratify patients for prevention and treatment, and to provide novel biomarkers that predict risk of exposure.

## Figures and Tables

**Figure 1:**
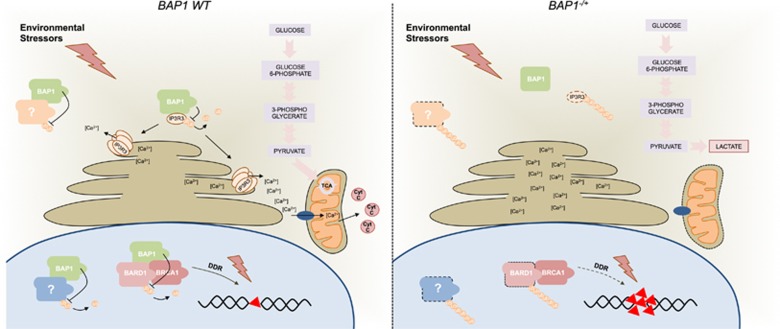
BAP1 is a ubiquitin C-terminal hydrolase belonging to the larger group of deubiquitylating enzymes. Germline mutations in *BAP1* gene identify a multi-cancer syndrome associated with onset of most types of malignancies and extremely high incidence of tumours related to environmental carcinogens, such as malignant mesothelioma (from asbestos), and uveal and cutaneous melanomas (from UV). The study by Carbone *et al.* describes a novel cytoplasmic function of BAP1. BAP1 controls ubiquitylation and stability of the endoplasmic reticulum (ER) Ca^2+^ channel, IP3R3. Deficiency of BAP1 is indeed associated to reduction of cytoplasmic Ca^2+^ upon pro-apoptotic stress and consequent reduced mitochondrial cell death. Concurrent impaired nuclear activity of BAP1 cooperates with the altered ER function resulting in resistance to apoptosis and promotion of tumour transformation. Overall this determines increased susceptibility to carcinogenesis. Residual expression of truncated forms of BAP1 can occur in BAP1^+/^^−^ cells; these are sequestered in the cytoplasm and lose interaction capacity with IP3R3. A second study by Carbone describes a peculiar metabolic rewiring of non-malignant BAP1^+/−^ cells. BAP1^+/−^ fibroblasts from carrier individuals display elevated aerobic glycolysis, high lactate production and reduced mitochondrial respiration. This metabolic profile is compatible with the ‘Warburg effect’ typically associated with malignancy. Whether and how this metabolic rewiring contributes to cancer predisposition in BAP1^−^^/+^ carriers will require further studies
